# The effect of aging on the repeated-dose liver micronucleus assay using diethylnitrosamine

**DOI:** 10.1186/s41021-022-00250-5

**Published:** 2022-08-18

**Authors:** Kensuke Satomoto, Isamu Suzuki, Koji Mita, Atsushi Wakita, Hiroshi Yamagata, Tatsuya Mitsumoto, Shuichi Hamada

**Affiliations:** Gotemba Laboratory, BoZo Research Center Inc, 1284 Kamado, Gotemba-shi, Shizuoka, 412-0039 Japan

**Keywords:** Micronucleus assay, Liver, Rat, Diethylnitrosamine, Ki-67

## Abstract

**Background:**

The repeated-dose liver micronucleus (RDLMN) assay has been well-developed and applied because of its simplicity and the ease of integration into general toxicity studies which is the preferred method from the 3R’s point of view. In this assay, we observed micronucleated hepatocytes which accumulated during a rather long-term dosing period. When considering integration into general toxicity studies, the effects of age of the animals used in the micronucleus assay becomes a major issue. The effect of age on the micronucleus induction rate has been reported in bone marrow micronucleus assays, and it is considered that the decrease in cell proliferation rate due to aging is the cause of the decrease in sensitivity. A decrease in sensitivity due to aging was also reported in a liver micronucleus assay using clofibrate and the cause is considered to be a decrease in hepatocyte proliferation activity due to aging. However, no actual decrease in hepatocyte proliferation rate due to aging has been reported. In addition, there are no reports, so far, on whether similar effects of aging appear when other substances were administered. To investigate the effects of aging in the RDLMN assay, this study focused on the effects of 14-day repeated administration of DEN, a well-known genotoxic hepatocarcinogen with the hepatocyte toxicity which should cause an elevation of cell proliferation rate as a reflective regeneration.

**Results:**

The liver micronuclei induced by DEN were equivalent between the two age groups (i.e., six and eight weeks of age at the start of dosing). In the histopathological examination for the liver, single cell necrosis, karyomegaly, and increased mitosis were observed in the hepatocytes, and the frequency and severity were increased dose-dependently. Ki-67 immunohistochemical analysis which can detect all cells in the cell cycle other than those in the G0 phase revealed dose-dependent increase of cell proliferation activity, and the difference between ages was not observed.

**Conclusion:**

The effect of aging on the RDLMN assay could not be recognized when DEN was administered for 14 days in rats. Meanwhile, it was supported by the histopathological examination and Ki-67 immunohistochemical analysis that such an effect of aging was masked by the compensatory hepatocyte proliferation which was induced by the hepatocyte toxicity of DEN.

## Introduction

The repeated-dose liver micronucleus (RDLMN) assay, which has been well-developed by the Mammalian Mutagenicity Study Group (MMS) in the Japanese Environmental Mutagenicity Society (JMES), is a useful in vivo test for the detection of chromosomal aberrations in hepatocytes. The turnover of hepatocytes is normally low, however, the accumulation of micronucleated hepatocytes (MNHEPs) by continuous treatment enables the evaluation of clastogenicity [1, 2]. It is assumed that the accumulation of MNHEPs is dependent on the proliferation activity of hepatocytes (HEPs). Hence, the age of subjected animals is an important factor in the RDLMN assay due to the higher proliferation rates of HEPs in younger animals. The RDLMN assay is designated to be incorporated into the general toxicity studies, especially in rats. On the premise of this usage, the Collaborative Study Group for the Micronucleus Test (CSGMT) of JMES/MMS conducted the RDLMN assay with 22 chemicals using only rats of six weeks of age at the start of dosing [2]. However, in some cases, the general toxicity study can be conducted with rats older than six weeks at the start of dosing [3]. Therefore, it has been pointed out that clarifying the effect of aging on the RDLMN assay is necessary [4]. A two-week RDLMN assay, based on six- and eight-week aged rats at the start of dosing, was conducted to examine the effect of aging on the result of the RDLMN assay. For this evaluation, diethylnitrosamine (DEN), which is a well-known genotoxic hepatocarcinogen with cytotoxicity in HEPs, was used. In addition, immunohistochemistry of Ki-67, an established cell proliferation marker which can detect all cells in the cell cycle other than those in the G0 phase, in HEPs was conducted using an anti-Ki-67 antibody for comparison of cell proliferation activity between the two groups of rats aged six and eight weeks at the start of dosing [5].

### Materials and methods

#### Animals

Male Crl:CD(SD) rats, which were five weeks of age at receipt and being six or eight weeks of age at the start of dosing, were purchased from The Jackson Laboratory Japan, Inc. (formerly Charles River Japan Inc., Tokyo, Japan). They were reared in an animal room that was controlled to maintain the temperature at 23 °C ± 3 °C, relative humidity of 50% ± 20%, air ventilation of 10 to 15 times per h, and 12-h illumination (7:00 a.m. to 7:00 p.m.). The animals, in groups of two or three, were housed in solid-floored plastic cages with bedding and given free access to pelleted diet in stainless-steel feeders, and tap water in water bottles. The animal experiments were conducted with the approval of the Institutional Animal Care and Use Committee and the test facility has been acquired Full Accreditation from the Association for Assessment and Accreditation of Laboratory Animal Care International.

#### Chemicals

DEN (CAS No. 55–18-5, > 99.0% purity) was purchased from Tokyo Chemical Industry Co., Ltd. (Tokyo, Japan), and diluted with physiological saline (Otsuka Pharmaceutical Factory Inc.). Physiological saline was used as the negative control substance.

#### Dose levels and treatment schedule

The rats (five males/group) were orally dosed with DEN at 0 (physiological saline), 3.13, 6.25, and 12.5 mg/kg for 14 consecutive days. The dosing period are long enough to detect a positive response in RDLMN assay and the dosage of DEN adopted in this study is enough to obtain a clear positive result in RDLMN assay [1]. The administration was performed by gavage using a flexible stomach tube at a dose volume of 10 mL/kg. The day of the first dosing was regarded as Day 1. All animals were observed daily for clinical signs and their body weights were recorded on Days 1, 7, and 14.

#### Liver micronucleus assay

The assay was performed according to the previous reports [1]. Briefly, 24 h after the last dosing, rats were euthanized by exsanguination via the abdominal aorta under anesthesia by isoflurane inhalation. The liver tissue was isolated from each animal and a part of the left lateral lobe (approximately 1 g) was cut out. The portion of the liver tissue was sliced at about 0.5 to 1 mm with a razor blade and washed well with Hank’s balanced salt solution (Thermo Fisher Scientific). The sliced liver was incubated in 20 mL of a collagenase solution (containing Collagenase Yakult: 100 U/mL, Lot No. 48001422, Yakult Pharmaceutical Industry Co., Ltd., Tokyo, Japan) at 37 °C for 1 h while being shaken at 50 rpm. After the treatment, the liver tissues and collagenase solution were mixed vigorously to isolate the HEPs. Subsequently, the mixture was filtered through a gauze and a cell strainer (pore size: 100 μm). Then, the HEP suspension was centrifuged at 50 × *g* for 2 min at 4 °C and the supernatant was discarded and 20 mL of 10% phosphate-buffered formalin was added to the pellet. The centrifugation was repeated and the supernatant was discarded again. Finally, the pellet was re-suspended in an equal volume of 10% phosphate-buffered formalin. Just before the microscopic observations, the HEP suspension was mixed with the same volume of a staining solution, which was a mixture of acridine orange (AO, 500 μg/mL) and 4',6-diamidino-2-phenylindole (DAPI, 10 µg/mL), and dropped onto a coverslip, which was then spread on a glass slide. The slide specimens were observed under a fluorescent microscope at 400 × magnification with U-excitation (ultraviolet ray excitation, wavelength: 330 to 385 nm).

#### Counting of micronuclei and mitotic phase cells

The number of MNHEPs in 4000 HEPs was counted for the sample from each animal, and the proportion of MNHEPs was calculated. In addition, the number of mitotic phase cells in 4000 HEPs was counted and the mitotic index (MI) was determined.

#### Histopathological assessment

The residual liver after sampling for micronucleus assay were fixed in phosphate-buffered 10% formalin. After fixation, liver tissue slices were embedded in paraffin, and sections were prepared and stained with hematoxylin and eosin (HE) for histopathological evaluation. Each lesion was graded according to its size.

#### Preparation of Ki-67 immunohistochemical specimens

The liver tissues were embedded in paraffin and sections were prepared. Then, immunohistochemical specimens were prepared as follows; briefly, tissue sections were deparaffinized in xylene, rehydrated in ethanol, and placed in a hydrogen peroxide solution to inactivate the endogenous peroxidase activity. Antigen retrieval was performed by microwave heating at approximately 98 °C for 15 min in the Target Retrieval Solution, pH 9.0 (Agilent Technologies, Santa Clara, CA, USA). After rinsing in phosphate-buffered saline, the sections were incubated with Ki-67 rabbit polyclonal antibody (Proteintech, Rosemont, USA) overnight at 4 °C. Visualization of immunolabeled antibodies was performed using EnVision + System-HRP Labeled Polymer Kit (Agilent Technologies, Santa Clara, CA, USA) following the addition of 3,3ʹ-diaminobenzidine/hydrogen peroxide as a chromogen. Sections were counterstained with Mayer’s hematoxylin, dehydrated, and covered with a glass coverslip.

#### Counting of Ki-67 positive cells

The Ki-67 immunohistochemical specimens were observed with a slide scanner (Aperio ScanScope XT, Leica Microsystems) and image processing software (Aperio ePathology Solutions, Leica Microsystems). The number of Ki-67 positive HEPs was counted and the area of the observed liver tissue was measured. From these parameters, the number of Ki-67 positive cells per unit area (/cm^2^) was calculated.

#### Statistics

For the body weight, MI, and the number of Ki-67 positive cells per unit area (/cm^2^), homogeneity of variance was analyzed by Bartlett’s test. When the variances were homogeneous, Dunnett’s test was applied to compare the mean value between the negative control group and each of the DEN-treated groups [6, 7]. When the variances were heterogeneous, Steel’s test was applied to compare the mean rank between the negative control group and each of the DEN-treated groups [8]. For the number of MNHEPs in 4000 HEPs, a pairwise comparison was conducted between the negative control group and each of the DEN-treated groups using the Fisher’s exact test [9]. In addition, dose-dependency was assessed using the Cochran-Armitage trend test [10–12]. These analyses were performed with the integrated statistical packages SAS Release 9.1.3 (SAS Institute Inc.) and EXSUS Version 7.6 (EP Croit Co., Ltd.). It has been confirmed in the test facility that the Fisher’s exact test can give the same statistical results as the Kastenbaum and Bowman’s method commonly used in LMN assay.

## Results

### Clinical signs and body-weight assessment

**Fig. 1:**
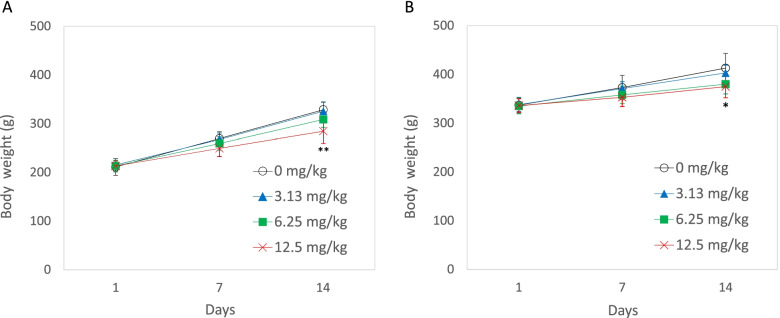
Body-weight changes in rats treated with DEN for 14 days. **A**: Six weeks of age at the start of dosing, **B**: Eight weeks of age at the start of dosing. The data are shown as mean ± SD (*n* = 5). *p < 0.05, **p < 0.01, significantly different from the negative control group

In the observations, no abnormal clinical signs were noticed in any animal throughout the dosing period (data not shown). The body-weight change was similar between the two age groups. At the doses of 12.5 mg/kg of DEN, the body weights were lower than the negative control group with a statistical significance in each group (Fig. 1A and B).

### Frequency of MNHEPs and MI

**Fig. 2:**
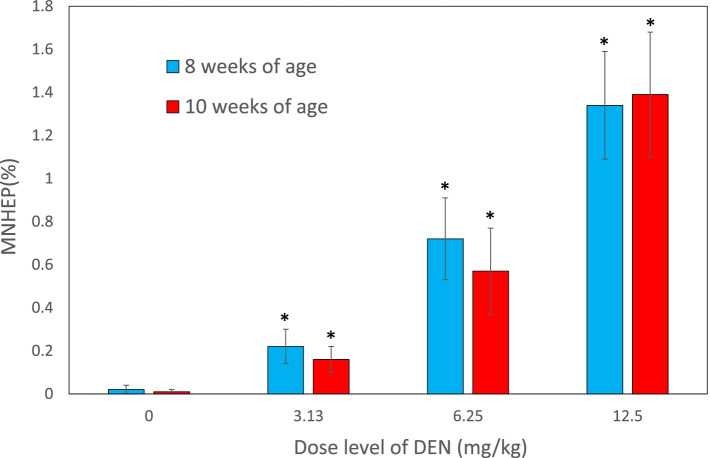
Frequencies of MNHEPs in rats of 8 or 10 weeks of age after treatment with DEN for 14 days. The data are shown as mean ± SD (*n* = 5). *p < 0.05, significantly different from the negative control group

**Fig. 3:**
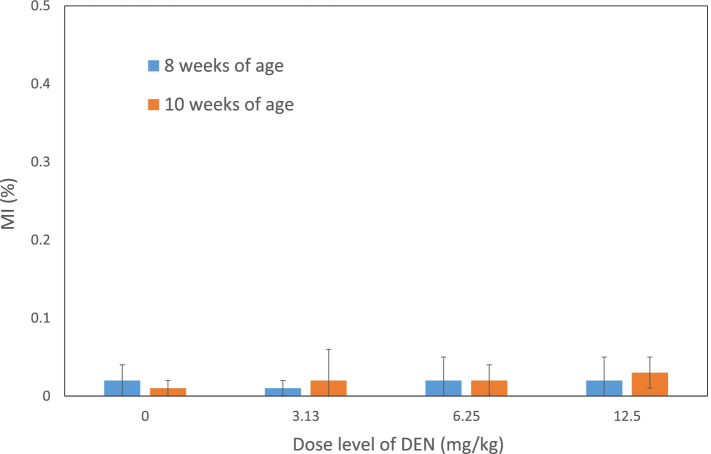
MI in rats of 8 or 10 weeks of age after treatment with DEN for 14 days. The data are shown as mean ± SD (*n* = 5). There were no statistical significant difference between the negative control group and each of the DEN-treated groups

In rats of 8 and 10 weeks of age at the end of dosing (6 and 8 weeks of age at the start of dosing), the frequencies of MNHEPs at 3.13, 6.25, and 12.5 mg/kg were significantly higher than the control group with dose-dependency (Fig. 2). There were no obvious differences between the two age groups. On the contrary, MI was approximately equivalent among all the groups (Fig. 3).

### Histopathological findings

Histopathological findings of the liver in rats of 8 and 10 weeks of age at the end of dosing (6 and 8 weeks of age at the start of dosing) are summarized in Table 1. In the liver, single cell necrosis and karyomegaly in the centrilobular area, and increased mitosis of the hepatocytes were observed in a dose-dependent manner. There were no obvious differences between the two age groups.

### Number of Ki-67 positive cells

**Fig. 4:**
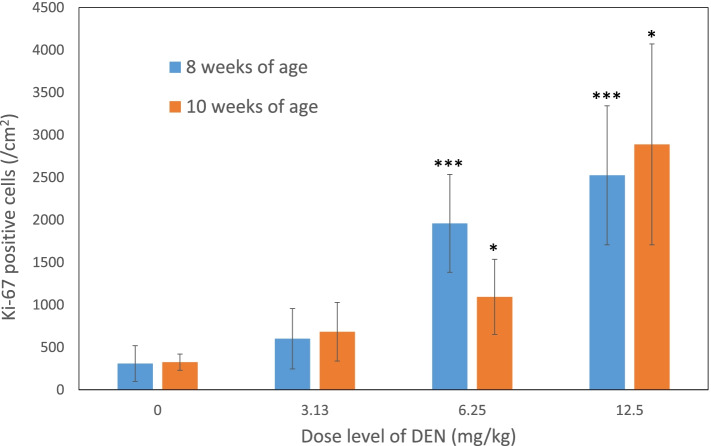
Ki-67 positive cells in rats of 8 or 10 weeks of age after treatment with DEN for 14 days. The data are shown as mean ± SD (*n* = 5). *p < 0.05, ***p < 0.001, significantly different from the negative control group

In rats of 8 and 10 weeks of age at the end of dosing, the numbers of Ki-67 positive cells at 3.13, 6.25, and 12.5 mg/kg were significantly higher than in the control group with dose-dependency (Fig. 4). There were no obvious differences between the two age groups.

## Discussion

The effect of aging on the micronucleus induction rate has been reported in bone marrow micronucleus assays with cyclophosphamide which induces bone marrow suppression by inhibition of DNA synthesis, and it is considered that a decrease in cell proliferation rates due to aging is the cause of the decrease in sensitivity [13]. In addition, a decrease in the sensitivity due to aging was reported in a liver micronucleus assay using clofibrate, an exogenous ligand of peroxisome proliferator-activated receptor alpha, and it was considered to be caused by a decrease in hepatocyte proliferation activity due to aging [14]. However, no actual decrease in hepatocyte proliferation rate due to aging has been reported. In addition, there are no reports, so far, on whether similar aging effects are observed when other substances are administered. Here, the effect of aging on the RDLMN with DEN, which is an alkylating agent and the clastogenicity and hepatocyte toxicity are well-known, was investigated [1]. For this investigation, two age groups were provided; one group was six weeks of age at the start of dosing and the other group was eight weeks of age at the start of dosing. Experimental animals in both groups were treated with DEN for 14 days. This investigation revealed that the frequencies of MNHEPs were equivalent between the two age groups and the effect of aging could not be delineated in the frequency of MNHEPs, contrary to the previous reports referenced above [13, 14]. It is assumed that this result was derived from the hepatocyte toxicity of DEN which was confirmed as necrosis in the hisopathological examination, which then accelerated the cell cycle as a reflective regeneration. Perhaps, the elevated proliferation activity enhanced the accumulation of MNHEPs and masked the effect of aging on the frequency of MNHEPs. Importantly, the dose-dependent increase in Ki-67 positive HEPs, which was much higher than that in the negative control group, showed compensatory hepatocyte proliferation sufficient to mask the slight age-related difference.

Not only proliferation activity but also a contribution of cytochrome P450 to the effect of aging described above also should be considered. Although, DEN has been reported to be activated by CYP2E1, an effect of the metabolic enzyme seems to be ignorable in the present experiment because the activity of CYP2E1 should not be different between the two age groups [15, 16]. The result of the present experiment led to an expectation that the liver micronucleus assay using a substance that strongly induces regenerative cell division due to its strong hepatocellular toxicity, such as DEN, is not significantly affected by aging. Although the proliferative activity of hepatocytes in the liver micronucleus assay had been evaluated by MI, evaluation by MI under the current conditions is considered to have extremely low sensitivity and cannot be properly performed. The low sensitivity of MI in the RDLMN has been also reported by CSGMT of JMES/MMS [2]. This time, as a result of evaluation based on the proliferative activity using an anti-Ki-67 antibody, it was confirmed that the proliferative activity of hepatocytes clearly increased in a dose-dependent manner in rats treated with DEN. This is probably based on the fact that MI is only an indicator of the proportion of M-phase cells, which is a temporary phase of the entire mitotic phase, while Ki-67 detects all cells in the cell cycle including G1, S, G2, and M-phase cells other than those in the G0 phase [5]. Summarily, the results of Ki-67 immunohistochemistry indicate the status of hepatocyte division more accurately than MI, which will be a very useful index for the evaluation of cell division in future liver micronucleus assays. Therefore, the evaluation based on Ki-67 immunohistochemistry is highly recommended.

## Conclusion

 The effect of aging on the RDLMN assay could not be recognized in rats treated with DEN for 14 days. On the other hand, it was confirmed that DEN induced histopathological changes including necrosis, karyomegary, and increased mitosis. In addition, Ki-67 immunohistochemical analysis revealed that the proliferation activity of hepatocytes clearly increased. These changes were observed in a dose-dependent manner with no age-related difference. From these results, it was concluded that DEN that strongly induces regenerative cell division due to its strong hepatocellular toxicity increased frequency of MNHEPs without being affected by aging in the 14-day RDLMN assay.

**Table 1:** Histopathological findings in the liver

Age at the end of dosing:		8 weeks				10 weeks			
Dose (mg/kg):		0	3.13	6.25	12.5	0	3.13	6.25	12.5
Findings	No. of animals examined:	5	5	5	5	5	5	5	5
Single cell necrosis, hepatocytes, centrilobular		0	4	5	5	0	3	5	5
minimal		–	4	3	–	–	3	2	–
mild		–	–	2	5	–	–	3	5
Karyomegaly, hepatocyte, centrilobular		0	3	5	5	0	3	5	5
minimal		–	3	2	–	–	3	2	–
mild		–	–	3	4	–	–	3	3
moderate		–	–	–	1	–	–	–	2
Mitosis, increased, hepatocyte		0	0	2	5	0	0	2	5
minimal		–	–	2	5	–	–	2	4
mild		–	–	–	–	–	–	–	1

–: no finding in this grade

## Data Availability

All data generated or analyzed during this study are included in this published article.

## Abbreviations

RDLMN: Repeated-dose liver micronucleus
DEN: Diethyl nitrosamine
MNHEP: Micronucleated hepatocyte
MN: Micronucleus
MMS: The Mammalian Mutagenicity Study Group
JMES: The Japanese Environmental Mutagenicity Society
CSGMT: Collaborative Study Group for the Micronucleus Test
HEP: Hepatocyte
AO: Acridine orange
DAPI: 4',6-Diamidino-2-phenylindole
MI: Mitotic index
PBS: Phosphate-buffered saline
